# Quantum random walks on congested lattices and the effect of dephasing

**DOI:** 10.1038/srep19864

**Published:** 2016-01-27

**Authors:** Keith R. Motes, Alexei Gilchrist, Peter P. Rohde

**Affiliations:** 1 Centre for Engineered Quantum Systems, Department of Physics and Astronomy, Macquarie University, Sydney NSW 2113, Australia; 2 Centre for Quantum Computation and Intelligent Systems (QCIS), Faculty of Engineering & Information Technology, University of Technology Sydney, NSW 2007, Australia

## Abstract

We consider quantum random walks on congested lattices and contrast them to classical random walks. Congestion is modelled on lattices that contain static defects which reverse the walker’s direction. We implement a dephasing process after each step which allows us to smoothly interpolate between classical and quantum random walks as well as study the effect of dephasing on the quantum walk. Our key results show that a quantum walker escapes a finite boundary dramatically faster than a classical walker and that this advantage remains in the presence of heavily congested lattices.

Quantum information processing^1^ promises many interesting technologies that are not available today. Perhaps most interesting is the promise for quantum computation, whereby quantum algorithms can be implemented that outperform their classical counterparts. The best known example is Shor’s factoring algorithm^2^, which can factor numbers exponentially faster than the best known classical factoring algorithm. Other examples include Grover’s database search algorithm^3^ and various graph theoretic algorithms^4^,^5^,^6^.

One route to implementing quantum information processing tasks is via quantum random walks^7^,^8^,^9^,^10^ whereby a particle, such as a photon, ‘hops’ between the vertices in a lattice. In this paper the effects of a congested, or obstructed, lattice on a quantum random walk (QRW) are studied and compared to a classical random walk (CRW). Congestion can be thought of as traffic and the walker is like a car trying to avoid the traffic. The quantum walkers also suffer a dephasing process as they propagate. This study provides insight into how random errors in the lattice and dephasing affect the dynamics of random walks and the robustness of certain quantum features. In our model, congestion refers to where the lattice through which the walker propagates has defects. These random defects are like blocked streets that the walker encounters and has to back out of during the next step. These defects are stationary during the evolution of the random walk, though we average over many such random lattices. Dephasing occurs when the state decoheres and is implemented via a dephasing channel acting after each step. In the limit of full dephasing the QRW becomes a CRW, so that dephasing also allows us to interpolate between the classical and quantum regimes. For an experimental demonstration of dephasing in a QRW see Broome *et al.*^11^, and for related theoretical work on QRWs with phase damping see Lockhart *et al.*^12^.

For characterising the resulting probability distributions for QRWs and CRWs we use variance and ‘escape probability’, that is the probability that the walker escapes a finite region of the lattice, or more picturesquely, the probability that the walker ‘beats the traffic’.

## Quantum Random Walks

A QRW describes the evolution of a quantum particle through a given topological structure represented as a *d* dimensional lattice. In a CRW, the walker probabilistically follows edges through a lattice to step to an adjacent vertex. In a QRW on the other hand, the walker spreads as a superposition of different paths through the graph. Physically, the walker can be a wide range of quantum particles, though of particular interest is the photon as photons are readily produced, manipulated and measured using off-the-shelf components in the laboratory. Photons have found widespread use in quantum information processing, most notably linear optics quantum computing (LOQC)^13^. These technologies provide the topological structure for implementing a QRW. They also allow for multi-photon QRWs^14^, which increases the dimensionality of the walk. For a further review on QRWs see refs 7, 8, 9, 10, and see refs 15, 16, 17, 18, 19, 20, 21, 22, 23 for the numerous optical demonstrations of elementary QRWs that have been performed.

## Quantum random walk formalism

To illustrate our QRW formalism we present the details for a one-dimensional discrete QRW on an unbounded lattice without any defects. The state of a one-dimensional QRW at any given time has the form,


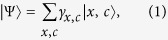


where 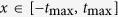 represents the position of the particle; 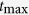 represents the total number of time steps and the size of the lattice; 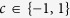 is the coin value that tells the walker whether to evolve to the left 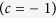 or right 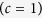; and 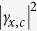 is the probability amplitude for a given position and coin value. The dimension of the lattice is 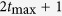. Since there are two coin values for each position, the probability that the walker is at position *x* is given by,


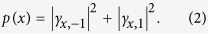


The one-dimensional walker begins at some specified input state 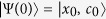 before it begins to evolve at time 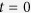, where 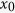 and 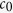 are the starting position and coin values respectively. The state then evolves for a finite number of time steps. The evolution is described by two operators: the coin 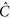 and step 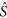 operators,


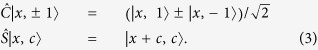


The coin operator takes a state and maps it to a superposition of new states using the Hadamard coin,


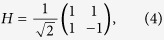


exploiting both possible degrees of freedom in the coin while maintaining the same position. Next, the step operator 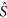 moves the walker to an adjacent position according to the value of *c*. 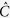 and 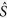 act on the state at every time step and thus the evolution of the system after *t* steps is given by,


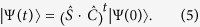


If the walker begins at the origin or on an even lattice position then, as the walker evolves, it lies on odd positions for odd time steps and on even positions for even time steps. Thus, as the walker evolves, the allowed locations for the walker oscillate between even and odd sites.

It is straightforward to generalise equation (1) to multiple dimensions by expanding the Hilbert space. For example, a two-dimensional walk would have the form,


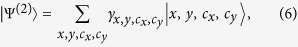


where 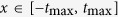 and 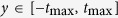 denote the two spatial dimensions, 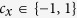 indicates for the walker to move left or right, 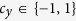 indicates for the walker to move down or up, and the superscript represents the dimension. The dimension of the two-dimensional system is 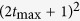. The coin and step operator can be generalised by taking a tensor product for each respective dimension, or alternately a coin could be employed which entangles the two dimensions. In the case of a spatially separable two-dimensional coin one obtains 
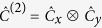
 and 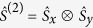. Likewise, the Hadamard coin for two dimensions becomes 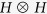.

After the system evolves, a measurement is made on either the position or the coin degree of freedom yielding the output probability distribution. With this probability distribution various metrics can be defined to characterise the evolution of the system, which we define next.

## Random Walk Metrics

The two common metrics that we use to quantify a QRW are the variance 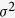 and the escape probability 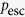. All simulations done in this paper have the initial condition that the walker begins at the origin 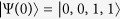. Also, all statistics are averaged over one hundred simulations unless the walk was deterministic in which case only one simulation was needed. Although the sample space is exponential in size, averaging over an exponential number of simulations is not feasible; however, one hundred simulations is sufficient for our work because it produces stable statistics that converge to fixed values and it smooths out the oscillations between data points.

## Variance

**Figure 1:**
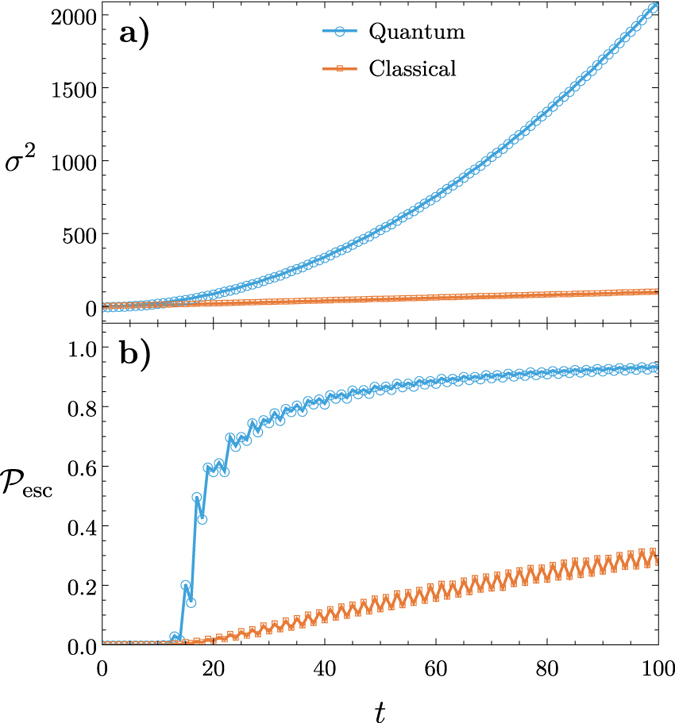
(**a**) The variance 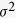 versus time *t* for the CRW and QRW on a two-dimensional square lattice defined by 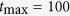. The rate of spreading is quadratic for the QRW and linear for the CRW. (**b**) The escape probability 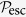 against time for the CRW and QRW on a two-dimensional square lattice defined by 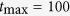 with a boundary defined by 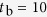. In the quantum case, the probability of escape is significantly larger for any given time after escaping than in the CRW.

The variance 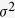 is a measure of how much the walker has spread out during its evolution. It is defined as,


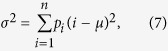


where 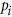 is the probability distribution of the walker, 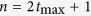 is the number of lattice sites, and 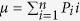 is the mean of the distribution. For calculating the variance in two-dimensions we take the variance of the marginal probability distribution where the probability distribution becomes 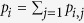 and 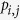 is the two-dimensional probability distribution. Figure 1a illustrates the variance versus time for both a QRW and a CRW on a two-dimensional square lattice of size 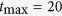. The QRW demonstrates a quadratic rate of spreading across the lattice while the CRW demonstrates a linear rate of spreading. This quadratic spreading is one of the distinguishing features of a QRW compared to the CRW. It forms the basis of some QRW algorithms such as the QRW search algorithm, which is quadratically faster than the corresponding classical algorithm. For simulations of the variance we don’t impose boundary conditions because the walker never reaches the boundary.

## Escape Probability

The escape probability 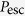 is a measure of how much of the walker’s amplitude leaks outside of a certain region in the lattice. To calculate 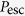 a boundary must first be defined which depends on the size of the lattice. For the square two-dimensional lattice we let the walker begin in the state 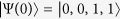 and let the escape boundary be two vertical lines at 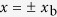, where 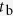 is the distance the escape boundary is from the origin 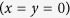. To calculate the escape probability on this square lattice we use,


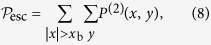


where 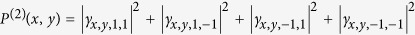 is the two-dimensional version of equation (2).

Figure 1b illustrates 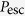 versus *t* for both a QRW and a CRW on a square lattice of size 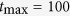 with a boundary given by 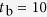. Here the QRW exhibits a dramatic jump in escape probability compared to the CRW. This is due to both the faster rate of spreading of the QRW, and to the QRW having larger amplitudes at the tails of its distribution. This dramatic jump is a key feature pointed out in this work that demonstrates an advantage that QRWs have over CRWs.

For all escape probability simulations the walker is allowed to walk back into the unescaped region which subtracts from the probability that the walker has escaped. This, in conjunction with the fact that the walker occupies alternating even and odd positions as the walker evolves, explains the oscillatory nature of the escape probability.

The two metrics, 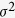 and 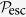, are closely related. If the walker has a large spread in its distribution then the walker also has a better chance to fall outside of the escape boundary. At any given time step *t* during the evolution we can determine the probability distribution over the lattice with equation (2) and then calculate these various metrics to be used for quantifying a random walk.

Any non-deterministic distribution obtained in this paper was obtained using a Monte-Carlo averaging technique. Since the sample space we are averaging over grows quadratically we are limited to about 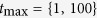 time steps. Next, we demonstrate how to add spatial defects, which cause congestion, into the walkers’ lattice and explore how the variance and escape probability are affected by this lattice congestion.

## Lattice Congestion

Lattice congestion is a model of defects in a medium. For the QRW and CRW the medium is the walkers’ lattice and the defects are modelled as blocked pathways where the walker has to enter the pathway to realise it is blocked and then reverse out on the next step. This model is closely related to percolation theory^24^ which models defects as missing lattice nodes. For a detailed introduction on percolation theory see^25^,^26^. Percolation is generally modeled on a *d* dimensional lattice with a given geometry such as a square, triangle or honeycomb. Regardless of geometry, the lattice consists of two components: *sites* and *bonds*. A site is a point on the lattice and a bond is the connection between the sites. These components give two strategies for introducing the random fluctuations that define percolation theory: *site percolations* and *bond percolations*. In site percolation the lattice sites exist with probability 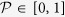 and when a site does not exist it is a defect in the lattice. In bond percolation the positions in a lattice are fixed while the bonds between the positions exist with probability *p*. The model in this paper is a variant of site percolation whereby the walker can occupy any site, but with probability 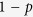 will find an obstruction and reverse direction upon hitting the respective site.

Percolation theory has an associated scaling hypothesis that predicts critical values, such as percolation thresholds^27^, which we do not reproduce in this manuscript due to our small lattice sizes. Instead we observe the behavior of QRWs on congested lattices and compare them to CRWs. However, we expect the same percolation characteristics such as percolation thresholds to exist in the underlying lattice that the walkers are exploring. For a two-dimensional square lattice with site percolations that most closely resemble the lattice used in this paper, the percolation threshold is 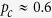
28. Values of 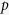 higher than this threshold produce long-range connectedness in the lattice. We make the comparison to percolation in this work because our spatial defects are equivalent to the defects in percolation theory; however, we do not observe the critical values that percolation theory predicts so we call it congestion to avoid confusion.

To generate a lattice with spatial defects a matrix of coin operators is constructed. The matrix is the same size as the lattice and each position in the matrix corresponds to a spatial position on the lattice. The coin operator corresponding to a given position then determines the behaviour of the walker. The coin operators are defined as either a Hadamard coin, equation (4), if the site is present, or a bit-flip coin,


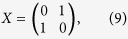


if the site contains a defect. For the two-dimensional case the bit-flip coin becomes 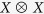.

As the quantum or classical walker evolves it will walk into these defects that signify congested points on the lattice. Upon reaching a defect the walker reverses direction, thus slowing the walker’s rate of spread. In this manuscript we define 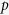 as the probability that the site is not a defect; therefore, the probability that a site is a defect is 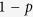.

## CRW on a congested lattice

The lattices we are considering contain randomly distributed defects, or points of congestion that impede the walker’s progress. Questions such as what is the probability that there is an open path from one side of the lattice to the other, are answered by *percolation theory*. There are many known applications for percolation theory^29^. A common example is asking whether a liquid can flow through a porous material. If enough pores (or sites) exist then the liquid can make it through. Another example is whether or not an electric current can flow through some medium where conductive sites are spread throughout some insulator. If enough conductive sites are present then a path will exist through the medium.

**Figure 2:**
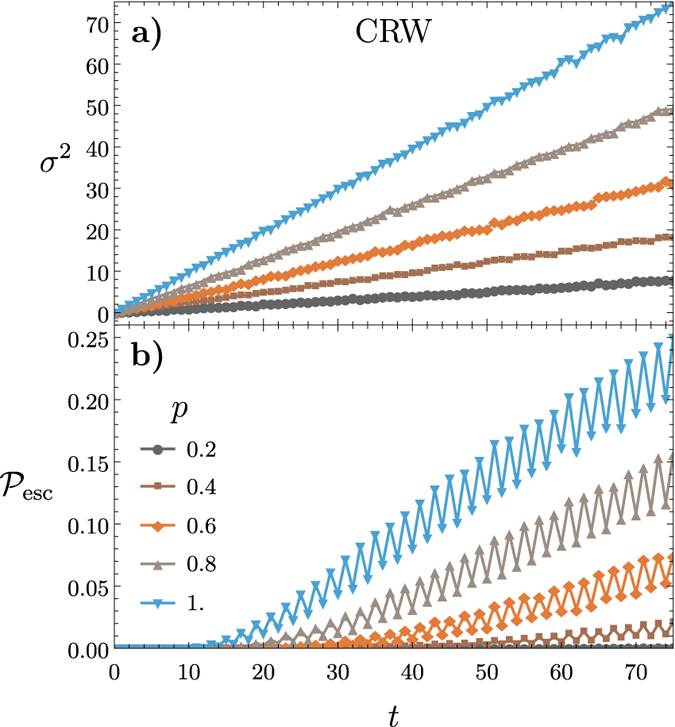
The variance *σ*^2^ and the escape probability 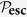 for a CRW plotted as a function of time *t* for varying congestion probabilities 1−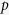 on a two-dimensional square lattice of size *t*_max_ = 75. (**a**) Reduced spreading is observed as congestion increases but the linear dependence remains. (**b**) The escape probability 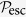 decreases as shown with an escape boundary of 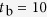. The walker tends to escape linearly with time.

Within the congested lattice we examine the spread of random walkers. Defects have the effect of reducing the rate of spread of the walker, or stopping it entirely if the lattice is so congested that there is no escape possible from the region the walker finds itself in. Figure 2a shows the variance 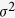 of a CRW versus time *t* in the presence of varying values of congestion 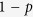 on a lattice of size 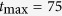. As the congestion increases the classical walker becomes trapped. In each case the variance preserves the linear dependence as is expected in a CRW.

In Fig. 2b the escape probability 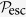 of a CRW is shown versus time *t* in the presence of varying values of congestion 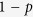 on a lattice of size 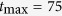 and escape boundary 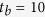. 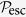 decreases as congestion is increased but remains linear modulo the oscillations being averaged out. Again there is a threshold where in terms of 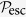 the walker stops escaping the boundary and the lattice becomes insulating.

## QRW on a congested lattice

Classically, the state can only move in one direction at a time while quantum mechanically the state spreads in a superposition of every direction simultaneously. As with a classical walker, the quantum walker escapes the bounded region more often if there are less defects. The significance of the quantum walker is both the quadratic spreading behaviour and the resulting probability distribution having more weight in the tails. For a review of work done on QRWs with percolation see^30^ for asymptotic results and analytic solutions. See^31^^,32^ for quantum tunneling effects on a one-dimensional QRW and, for a two-dimensional lattice, average distance measures and the order of quadratic scaling. This manuscript is unique from these two for several reasons. First, properties of a QRW on congested lattices with the 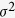 and 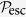 metrics were not studied in the previous two. Second, we compare QRWs to CRWs and observe whether QRWs maintain their advantages over CRWs on congested lattices. Third, we tune the random walks on congested lattices between being fully quantum and fully classical using a dephasing process, described later in this manuscript, which acts as an error model.

**Figure 3:**
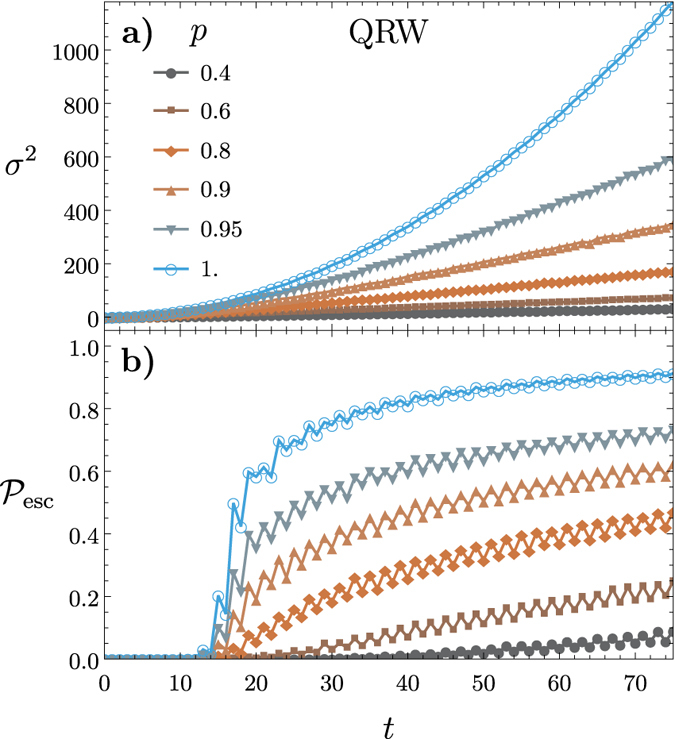
The variance *σ*^2^ and the escape probability 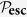 for a QRW plotted as a function of time *t* for varying congestion probabilities 1−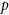 on a two-dimensional square lattice of size *t*_max_ = 75. **** (**a**) Reduced spreading is observed as congestion increases but the QRW maintains its advantages over CRWs with congestion. (**b**) The walker quickly escapes the boundary as compared to the classical walker. As 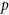 decreases the jump in 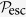 becomes less prominent as shown for an escape boundary of 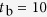.

Figure 3a shows the variance 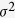 versus time *t* for a QRW with varying values of congestion 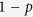 for 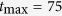. As congestion increases the variance of the walker decreases; however, it retains its quadratic (i.e. ballistic) spreading albeit with a different quadratic coefficient. This property shows that QRWs remain advantageous over CRWs even in the presence of lattice defects.

Figure 3b shows the escape probability 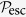 versus time *t* for varying values of congestion probability 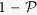 on a lattice of size 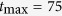 and boundary 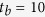. For 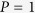 there is no congestion present and the 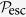 metric experiences a sudden jump from 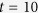 to 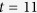. This is because the QRW has most of its amplitude in its tails as it evolves. When 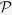 decreases and the lattice becomes more and more congested the sudden jump is still present at the same value of *t* but with a much smaller amplitude. This shows that QRWs retain their advantage over CRWs in the presence of heavy congestion. Note that the percolation threshold is around 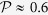, below which we expect that on average there is no clear route across the graph.

## Varying escape boundary

**Figure 4:**
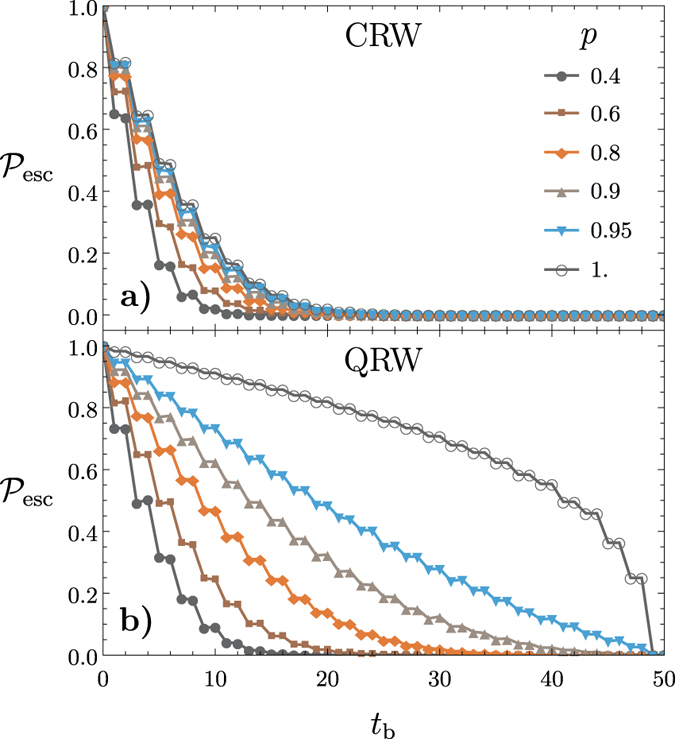
The escape probability 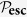 versus a varying escape boundary *t*_b_ for several values of congestion 1−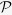 for the CRW (a) and the QRW (b). The walker evolves for 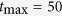 time steps. The QRW maintains a significantly larger 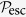 as the boundary moves away from the initial starting position than the CRW. In both cases 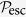 goes to zero as the boundary approaches the end of the lattice.

In the previous simulations involving escape probability the escape boundary was set to be near the initialised position of the walker. The next topic we consider on a congested lattice is how the escape probability on a congested lattice changes as 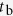 varies. Consider Fig. 4 which shows 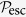 as a function of 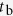 with varying values of congestion 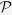 for the CRW (a) and the QRW (b). Both walkers evolve for 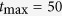 steps and 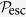 is calculated at 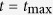. In both the CRW and QRW 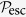 reduces with increased congestion and when 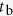 is farther from the walkers initial position. What is interesting is that the QRW maintains a significantly larger 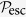 than the CRW as the escape boundary moves away.

## Dephasing

Next, we consider what happens to a QRW subject to dephasing. Dephasing represents decoherence caused by the environment which can be related to measurement errors caused by thermal fluctuations, white noise, photons interfering with the quantum walker, etc. To explore this we first introduce a model of dephasing and characterise it with our two metrics: variance and escape probability.

Consider a QRW where after each step, each state in the basis has probability 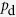 of acquiring a *π* phase flip. We can model this process as choosing to apply one of a set 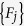 of unitary matrices covering all the combinations of ±1 on the diagonal. If 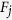 has *s* −1’s on the diagonal we choose it with probability 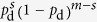.

The probability of a particular sequence will be the product of the probabilities of the 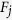 appearing in the sequence since they are independently chosen at each step. If 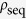 is the final pure density matrix appearing with probability 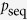, then in general the final state of the system is described by,


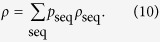


That is, for any POVM element 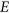 we have,


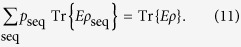


We algorithmically implement dephasing by randomly flipping the signs of individual kets in the walker’s superposition state with probability 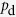, and average the results of any measurement at the end of a large number of runs. This in effect samples from the distribution represented by 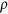 and is automatically weighted by the probability of a given sequence.

That this whole process represents dephasing is not immediately obvious. To see it, we first rewrite 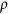 as the vector 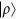 using the *vec* operation which simply stacks its columns on top of each other. Using the identity 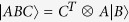 for any three square matrices *A*, *B*, and *C*; then grouping the terms that turn up, we can write,


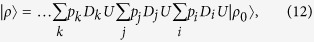


where 
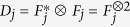
, *U* represents the step and coin operations, and 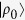 is the vectorised initial density matrix. This shows that after each step we apply the process described by the dynamical matrix,


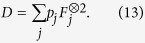


The matrices *F*_*j*_ are diagonal so we write the diagonal as a vector denoted by 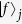, so that the diagonal of 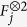 is 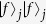. Since 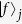 has only real entries we can rearrange it into the matrix 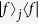. We can do a similar arrangement with *D* so that,


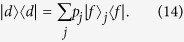


It’s worthwhile pausing and noting what this matrix represents. From equation (12) we can see that the diagonal of *D* multiplies the elements of the vectorised 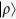. Hence when we arrange the values into a matrix, the entries of 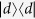 multiply the corresponding entries in *ρ*.

The first thing to note is that this matrix is symmetric. We will denote the entries of 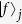 by 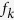 and drop the reference *j* for clarity. The diagonals of 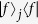 are of the form 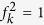 and since 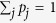 the diagonal of 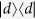 is unity and the process does not change the amplitudes of the states. The off-diagonals are of the form 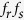 where 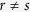 and their sum over *j* has the value,


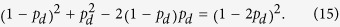


The terms on the left are the probabilities that both 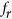 and 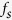 are positive, both negative, or one of each respectively. Each of these terms is multiplied by the binomial sum of the probabilities of all the combinations of ±1 on all the other elements of 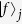 and not *r* or *s*, which evaluates to 1. Note that this result holds for any dimension. In summary, the map that is performed by *D* multiplies every off-diagonal element of *ρ* by 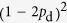. This is a dephasing map.

If 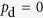 none of the signs are flipped, and if 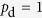 all of the signs are flipped. Since the QRW is invariant under a global phase flip, these two extremes reproduce an ideal QRW. When 0 < *p*_d_ < 1 dephasing is introduced into the system. A value of 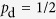 corresponds to complete dephasing which causes the walker to behave classically. The classical results in this paper were produced by using our QRW code with a value of 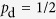. This was checked with purely classical code to verify that we are indeed obtaining a CRW.

If we imagine an inefficient measurement of the QRW at every step where it is projectively measured with probability 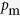 or otherwise left alone, this map would describe dephasing by a dynamical matrix which multiplies all the off diagonal elements of *ρ* by 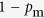. So our dephasing process is equivalent to a measurement performed with a probability 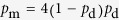.

**Figure 5:**
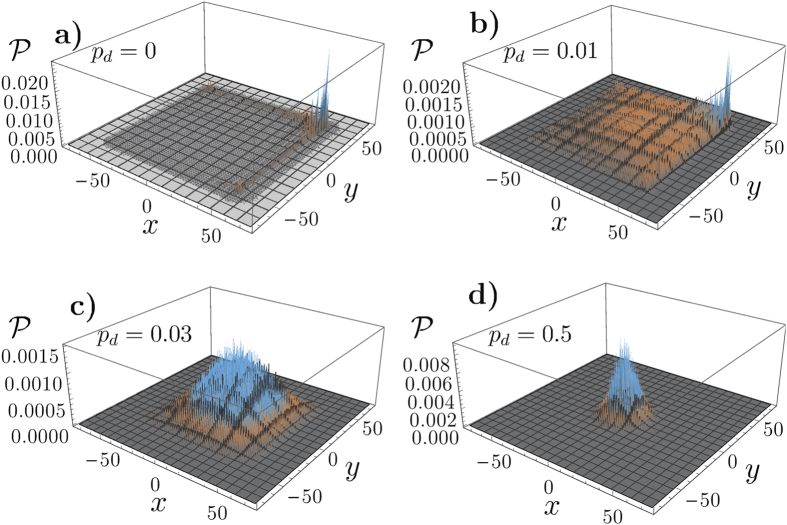
The QRW probability distribution shown at the final time step over a two-dimensional square lattice defined by *t*_max_ = 75 with no defects present. (**a**) The QRW with no dephasing 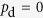 always yields a deterministic probability distribution with ballistic spreading. (**b**) The same QRW but with a dephasing probability of 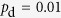. It has a similar probability distribution but begins approaching classical statistics. (**c**) The same QRW again but with a dephasing probability of 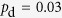. Here the probability distribution becomes centred around the origin which and begins to look much like the statistics of a CRW. (**d**) The same QRW again but with a dephasing probability of 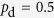. This is maximal dephasing and the walk become identical to a CRW.

In this work, dephasing is a method for introducing quantum decoherence to the QRW. To illustrate the effect of dephasing in our model we plot the probability distribution at the final time 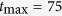 of various random walks in Fig. 5. In Fig. 5a the walk has no dephasing 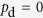 and is thus completely deterministic. We see that this probability distribution has one main peak near the positive *x* and positive *y* direction, which is in the initialised direction of the coins, and is at the edge of the lattice. This is in contrast to what occurs when dephasing is introduced. Figure 5b shows the same evolution again but with a dephasing probability of 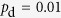. With this value of dephasing the distribution retains most of its quantum behaviour. Figure 5c shows the same evolution again but with a dephasing probability of 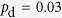. With this value of dephasing the probability distribution loses much of its quantum behaviour and begins behaving like a CRW. Finally in Fig. 5d we show the same evolution but with 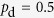 and obtain the probability distribution of a CRW.

We notice that with sufficiently strong dephasing the probability distribution becomes localized around the origin so that the QRW behaves like a CRW distribution. Note that the corresponding value of 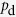 that collapses the QRW to a CRW depends on 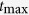. As 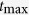 increases the underlying lattice has more sites where dephasing can occur and thus a smaller 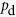 will cause the corresponding collapse. By incrementing 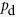 we can smoothly interpolate between QRWs and CRWs, which is a key feature of this work.

## Congestion & Dephasing Combined

**Figure 6:**
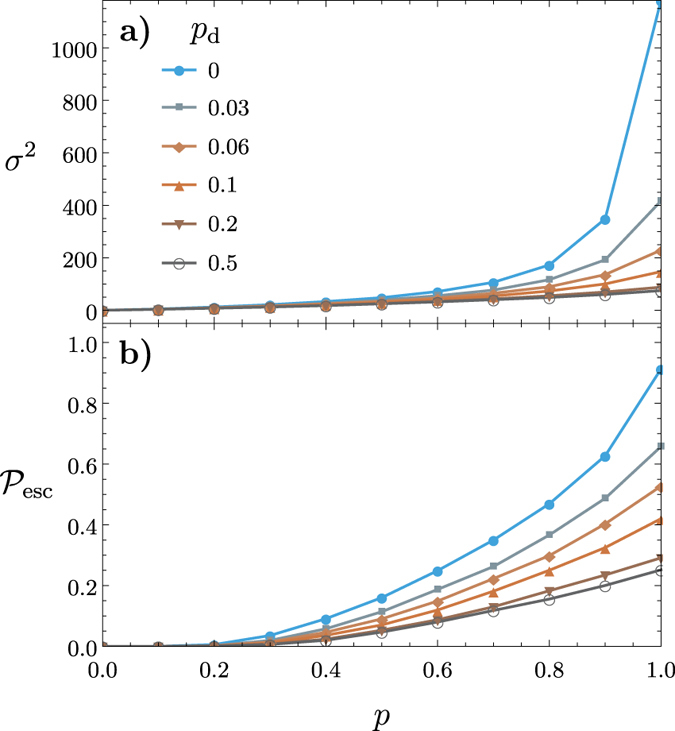
The variance *σ*^2^ and escape probability 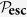 obtained at the final time step plotted against the congestion probability 1−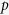 for varying values of the dephasing probability *p*_d_ on a square two-dimensional lattice of size given by *t*_max_  = 75. (**a**) The propagation of the walker decreases monotonically with the congestion rate for increasing values of dephasing 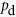. A quadratic behavior remains for small values of 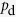. (**b**) The escape boundary is at 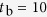. With decreasing 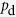 the quantum walker has a larger chance to escape the boundary. As 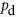 increases the QRW enters the classical regime and quantum advantages are lost.

Next we combine congestion and dephasing and examine the joint effects. Figure 6a shows the variance obtained at the final time step of the QRW as a function of the congestion probability 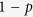 for varying values of the dephasing probability 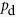 on a two-dimensional square lattice of size given by 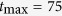. A monotonic decrease is observed in the variance for a given 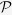 as 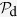 is increased and a quadratic rate of spreading is maintained for small values of 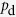. Figure 6b shows 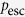 with boundary 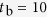 as a function of congestion probability 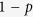 for varying values of dephasing probabilities 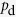 on a two-dimensional square lattice defined by 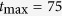. When 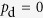 the walk is fully quantum so more of the probability distribution escapes the boundary. When dephasing is increased process errors are introduced, reducing 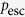 for any given value of *p*.

## Discussion

Quantum random walks are a promising route towards quantum information processing, exhbiting many unique features compared to the classical random walk. In the classical context, walks on percolated lattices (i.e. lattices containing congestion) have been well studied. We have considered the analogous situation in the quantum context. We defined a mapping between quantum and classical walks, via the coin operator, to allow for a direct comparison of the two. Then we introduced a model for adding static defects to the underlying lattice via the introduction of bit-flip coins. These defects inhibit the spread of the classical and quantum walker, reducing the escape probability and variance metrics. We found that as a quantum random walk evolves it will suddenly and dramatically escape a finite boundary. It maintains this property even in the presence of congestion.

We also introduce a dephasing error model. Dephasing errors are errors caused by the environment on the quantum walker as it evolves. In the limit of large dephasing the quantum random walk spatially localises and behaves like a classical random walk. The spread of the walker is sensitive to small amounts of dephasing in our dephasing model and becomes more sensitive as the size of the lattice increases.

We also studied the effects of spatial defects and dephasing together on the propagation of the walker and found a monotonic decrease is observed in the variance and escape probability for a given congestion probability as the dephasing probability is increased. Our results indicate that a quantum walker on a lattice with defects and dephasing still exhibit a quadratic rate of spreading. Thus, as the quadratic spread of quantum walks is one of the key features that make them applicable to quantum information processing applications, such as the quantum search algorithm, quantum walks on congested lattices remain advantageous over classical random walks.

## Additional Information

**How to cite this article**: Motes, K. R. *et al.* Quantum random walks on congested lattices and the effect of dephasing. *Sci. Rep.* **6**, 19864; doi: 10.1038/srep19864 (2016).
